# The Effects of Exercise on Acute Immune Responses in Relative Leisure-Deprived People Living with HIV/AIDS: A Pilot Study

**DOI:** 10.3390/ijerph19138155

**Published:** 2022-07-02

**Authors:** Xin-Min Qin, Ji-Young Park, Bo-Ram Kim, Chang-Hwa Joo

**Affiliations:** 1 Department of Sport Science, Kangwon National University, Chuncheon 24341, Korea; qxm@kangwon.ac.kr (X.-M.Q.); justdoit4878@gmail.com (J.-Y.P.); 2 Department of Physical Education, Korea University, Seoul 02841, Korea

**Keywords:** exercise, HIV/AIDS, immune responses, relative leisure deprivation

## Abstract

Exercise training involving exercises of optimal intensity and duration improves psychological and medical variables in relative leisure-deprived people living with HIV/AIDS. This study aimed to analyze associated psychological variables and the effect of exercise intensity and duration on immune responses in relative leisure-deprived people infected with HIV. The participants completed different moderate-intensity exercises (30 min (60–80% HR_max_) and 45 min (60–80% HR_max_)) and high-intensity exercise for 10 min (>80% HR_max_). Levels higher than “normal” were rated for relative leisure deprivation, indicating relative deprivation of leisure among participants. The overall level of quality of life was “normal”, indicating that quality of life was not considered high. The stress level was psychologically considered low. Time had a significant effect on cortisol levels (*p* < 0.05). Compared to pre-exercise, cortisol level was significantly decreased immediately after moderate exercise for 45 min and 3 h post-exercise after high-intensity exercise for 10 min (*p* < 0.05). However, time and the interaction of condition and time had no significant effect on IL-6 and sIgA levels (*p* > 0.05). Despite the small sample size of this pilot study, the results demonstrate that moderate-intensity exercise can be recommended to improve the health and quality of life of people infected with HIV.

## 1. Introduction

The epidemic-induced spread of viruses, such as the severe acute respiratory syndrome coronavirus 2, which causes COVID-19, does not only pose a challenge in the country where it originated but also causes unexpected global damage that has never been experienced in a social, economic, and cultural context. Among viral infectious diseases, Acquired Immune Deficiency Syndrome (AIDS), which is also called the “modern black death” and is recognized as a life-threatening disease, is caused by Human Immunodeficiency Virus (HIV) infection, resulting in a decrease in the body’s immune function or even death in the worst case [1]. Since HIV/AIDS was first reported in the United States in 1981, it has spread rapidly worldwide, and according to a World Health Organization (WHO) report, the number of people infected with HIV is approximately 37.7 million globally [2]. The number of Koreans infected with HIV and new infections was 12,991 (male: 93.2%, female: 6.8%) and 989 (male: 95.6%, female: 4.4%) in 2018, respectively [3]. Although the incidence of new infections has been steadily increasing since HIV-infected people were first reported in 1985, it is estimated to be more than five times higher than the reported figure, considering cases of infection unawareness due to the asymptomatic period and infected persons who do not report their infection due to social stigma and discrimination [4].

HIV infection occurs through blood-borne transmission and the vertical route of infection via childbirth among HIV carriers [5]. HIV infection directly or indirectly destroys CD4+ T cells, causing problems in the body’s immune system [6]. Since the introduction of highly active antiretroviral therapy, HIV/AIDS has been recognized as a chronic disease that can sustain life in the long term without immediate progression to fatal disease, provided the appropriate medication and health management are administered [7]. Antiretroviral therapy reduces the progression to AIDS due to various effects, such as a reduction in the HIV-related inflammatory response and an increase in the CD4 T-cell response [8,9]; however, it does not completely prevent a decrease in numbers and a decline in immune function [10]. This immune system dysfunction increases the inflammatory response and consequently causes oxidative stress, dyslipidemia, lipodystrophy, bone demineralization, and cardiovascular dysfunction [5].

Regular exercise improves not only psychological variables (quality of life and stress resistance, among others) but also medical variables (immune function) [11,12,13]. People infected with HIV have a lower quality of life than the general public because of a lower socioeconomic level, self-efficacy, and life satisfaction [14]; they are negative towards participation in exercise training and exhibit high levels of chronic fatigue and anger [15]. However, various studies have shown that regular exercise has a positive effect on improving the quality of life and lowering the premature mortality rate by improving mental health, physical fitness, and immune function in people infected with HIV, without any side effects [16,17]. Most studies on the effectiveness of exercise programs for people infected with HIV have been conducted in Africa, Europe, and North America [18]. However, to the best of our knowledge, no study has yet assessed the association of psychological factors, such as relative deprivation of leisure, quality of life, and stress level, with immune system response to exercise in people infected with HIV in Korea, where the number of new infections continues to increase annually.

Regular exercise is effective not only in improving physical strength but also in treating and preventing various diseases, such as cardiovascular disease, diabetes, and osteoporosis [12,19]. The effect of regular exercise can result from adaptation due to biochemical changes in the body that occur after acute exercise [20]. Biochemical changes that induce adaptation to physical activity may occur during exercise and persist for a few minutes, hours, or even days after exercise. The degree and duration of biochemical changes during an acute response can vary depending on the intensity and duration of exercise [21,22,23,24,25,26]. Many studies have analyzed the effects of regular exercise on the immune system, physical fitness, and quality of life, among others; however, conflicting results have been obtained regarding the same study variable [14,27]. There may be various reasons for these contradictory results. The main reason could be that the effect of exercise training comprising a single exercise type was analyzed without considering the comparative group, race, and life characteristics. This is because recruiting people infected with HIV as research participants is more difficult than recruiting from the general population [28]. Considering these research limitations, further research is required to determine the most effective exercise intensity and duration before proceeding with a study that analyzes the effect of long-term exercise training on people infected with HIV. Therefore, despite the difficulty associated with recruiting participants, the present pilot study focused on analyzing associated psychological variables and the effect of exercise intensity and duration on immune responses in relative leisure-deprived people infected with HIV.

## 2. Materials and Methods

### 2.1. Participants

Twelve male patients infected with HIV who had been screened for HIV with positive results were recruited through the Korean Association for AIDS Prevention Center and volunteered to participate in the study. Three participants withdrew from the study during the experiment because of difficulties performing high-intensity exercise; hence, nine people participated in all the experiments. Participants participated in the study after receiving a full explanation of the protocol and providing informed consent. Ethical approval was granted by the Ethics Committee of Kangwon National University (KWNUIRB-2021-09-003-003).

### 2.2. Study Design

**Figure 1:**
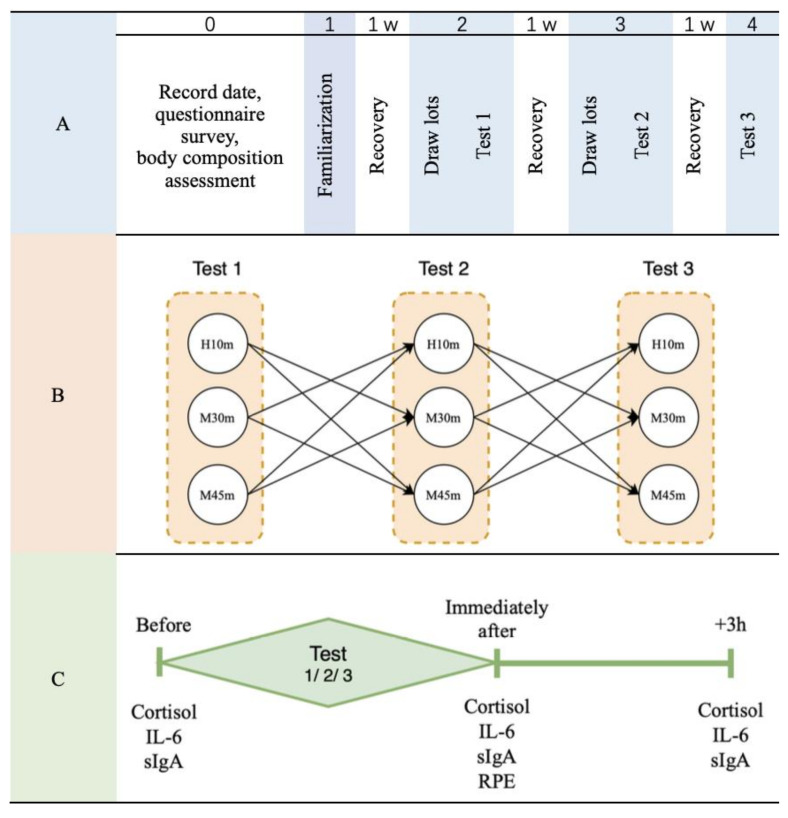
Experimental group assignment. (**A**): experiment; (**B**): exercise trial; (**C**): sample collection; 1 W: a week; H10m: high-intensity exercise for 10 min (>80% HRmax); M30m: moderate-intensity exercise for 30 min (60–80% maximum heart rate [HRmax]); M45m: moderate-intensity exercise for 45 min (60–80% HRmax); Before: before the exercise; Immediately after: immediately after the exercise; +3 h: 3 h post-exercise; IL6: Interleukin-6; sIgA: saliva Immunoglobulin A; RPE: rate of perceived exertion.

The experiment was conducted at a fitness center. Before the experimental session, the participants visited the laboratory where the project was performed and its details were explained: (1) the following data were recorded: comorbidities, drug use, smoking, and dietary habits; (2) the questionnaire was completed; and (3) body composition was assessed (Inbody 470, InBody Co., Ltd., Seoul, Korea). The first session was performed to familiarize participants with the test environment, exercise trial, and safety issues involved in the experiment. Sessions 2 to 4 are the experimental stages (Figure 1A).

In this study, the intensity and duration of exercise were established based on a previous study [26]. To analyze the effect of time difference in moderate-intensity exercise, two forms of exercise were selected. Because of the low physical fitness of participants, high-intensity exercise consisted of short-term exercise rather than long-term exercise. The exercise trial was classified into three tests, each with different intensity and duration of exercise. The content of the three tests includes (1) moderate-intensity exercise for 30 min (60–80% maximum heart rate [HR_max_]; M30m) and 45 min (60–80% HR_max_; M45m) and (2) high-intensity exercise for 10 min (>80% HR_max_; H10m). Each person selected one test at a time, repeated the exercise trials three times, and had at least one week of recovery time after the experiment to prevent the previous experiment from influencing the next experiment (Figure 1B).

For all participants, the exercise during the first two tests was randomly determined by drawing lots, and the content of the third test was determined based on the first two tests (test 1: three different exercises were put inside the same envelope, and the participants randomly selected one; test 2: the method of selection in test 1 was repeated for the remaining two test contents; test 3 is the remaining content after the selection of the first two choices); the contents of the three tests were not repeated (Figure 1A,B).

### 2.3. Study Procedures

When the participants arrived at the test site, they changed into sports clothes, and we collected saliva samples from them before exercise after a full rest. The participants had previously been instructed to clean their teeth after the last meal on the day preceding the examination. Thereafter, the participants underwent the exercise protocol selected on a random heart rate monitor. The trial was performed between 9:00 a.m. and 10:00 a.m.; heart rate was recorded every 2 min during exercise. Saliva samples were collected, and the rate of perceived exertion (RPE) was checked immediately after exercise. After that, participants sat down and rested at room temperature (23 ± 2 °C) for 3 h, and their saliva samples were collected again (Figure 1C).

### 2.4. Exercise Trial

Participants started running on the treadmill at an initial speed of 3 km/h for 3 min, and the running speed was increased by 1.0 km/h at 1 min intervals. The real-time HR was tracked and recorded using a Polar watch (Polar WearLink^®^; Polar Electro, Kempele, Finland). The test commenced when the HR reached the target (moderate-intensity exercise: 99.3 ± 6.5 beats; high-intensity exercise: 132.4 ± 8.7 beats). The treadmill speed was adjusted according to the real-time HR based on a Polar watch to maintain the target HR and achieve the target exercise intensity (moderate-intensity exercise: 5.52 ± 1.49 km/h; high-intensity exercise: 6.92 ± 1.70 km/h). Only full-target time periods were considered complete. To ensure experimental rigor and avoid confusion, participants who failed to complete the entire test or reach the target HR and target time during the test were excluded.

### 2.5. Sample Collection and Analysis

Saliva was collected using 1 mL plastic Salivette^®^ collection tubes (Sarstedt Inc., Nümbrecht, Germany), immediately frozen, and stored at −70 °C until biochemical analysis. Saliva samples were collected pre-trial, immediately post-trial, and 3 h after the exercise from each trial, with each participant providing three saliva samples at a time. No participant was allowed to eat or drink before saliva sample collection. Secretory IgA (sIgA), interleukin-6 (IL-6), and cortisol levels in the saliva were measured by enzyme-linked immunosorbent assay using a Salimetrics^®^ kit (Salimetrics LLC, State College, PA, USA).

### 2.6. Questionnaires

The relative leisure deprivation scale was based on a questionnaire used in previous studies [29]. It comprises 18 items with four subfactors as follows: egoistical deprivation, resourceful deprivation, cognitive deprivation, and emotional deprivation. Regarding the quality of life, the Korean version of the WHO Quality of Life-HIV Brief developed by the WHO in 2002 was used; it consists of six areas and a total of 31 questions. The stress level was rated using a questionnaire comprising 20 items and the three following subfactors: exhaustion, depression, and anger. In addition, the survey tools were measured on a 5-point Likert scale ranging from “strongly disagree” (1 point) to “strongly agree” (5 points). RPE was recorded using the 20-point scale developed by Swedish researcher Gunnar Borg (6: no exertion at all; 7.5 extremely light; 9: very light; 11: light; 13: somewhat hard; 15: hard; 17: very hard; 19: extremely hard; 20: maximal exertion), to estimate perceived exertion after exercise [30].

### 2.7. Statistical Analysis

Statistical analysis was performed using SPSS 26.0 (version for MAC; Chicago, IL, USA). Means and standard deviations (mean ± standard deviation [SD]) were calculated for all variables. Descriptive statistical analysis was used to analyze the psychological variables. Comparisons of sIgA, IL-6, and cortisol levels before, after, and 3 h after different-intensity exercises were made using a mixed-model two-way repeated-measures analysis of variance (ANOVA) (3 intensity × 3 group) with planned contrasts at different intensity points. When a significant effect was observed, differences among time points were examined using a repeated-measures one-way ANOVA. The results for continuous variables are presented as the mean ± SD, and statistical significance was set at *p* < 0.05.

## 3. Results

### 3.1. Demographic Characteristics

The demographic characteristics of the participants are presented in Table 1. Regarding marital status, 77.8% of participants were unmarried, 22.2% were divorced, and none were married or widowed; simultaneously, 88.9% had no children. More than half of the HIV-infected individuals were unemployed, accounting for 55.6% of the total participants. Concurrently, 33.3% were engaged in public services, and only 11.1% were self-employed. In addition, 66.7% of the patients lived in rural areas, whereas 33.3% lived in cities.

The education and income levels of the participants were low. Only 33% of the participants had graduated from college or university, and 78% earned a monthly income of <800 dollars and lived on the basic pension provided by the government. As for the duration of HIV infection, 55.6% had been infected for over 10 years, 33.3% for 6–10 years, and only 11.1% for 0 years. In addition, most of the respondents were asymptomatic, accounting for 66.7% of the total participants, 11.1% were symptomatic, and 22.2% were in the stage of AIDS.

### 3.2. Relative Leisure Deprivation

Relative leisure deprivation is distributed from the lowest score of 1 to the highest score of 5, and a higher score indicates higher relative leisure deprivation. Among the subfactors of relative leisure deprivation, the level of resource deprivation was found to be the highest, followed by cognitive, personal selfish, and emotional deprivation (Table 2). Levels higher than “normal” were rated for most factors, indicating relative deprivation of leisure among participants.

### 3.3. Quality of Life

Among the subfactors of quality of life, the overall level was “normal”, indicating that quality of life was not perceived as high (Table 3).

### 3.4. Stress Level

Perceived stress, including burnout, depression, and anger, exhibited low indices, suggesting that stress level was perceived as low psychologically (Table 4).

### 3.5. Salivary Cortisol, IL-6, and sIgA Indices in Different Exercise Protocols and Time

Time had a significant effect on cortisol levels (*p* < 0.05) (Table 5). Compared to pre-exercise, it was significantly decreased immediately after exercise in M45m and at 3 h post-exercise in H10m (*p* < 0.05). However, time and the interaction of condition and time had no significant effect on IL-6 and sIgA levels (*p* > 0.05). Although the cortisol levels of the exercise-induced variables were higher than the reference values of the previous study, the IL-6 and IgA values were comparable [31,32,33].

### 3.6. Heart Rate and RPE

During moderate-intensity M30m and M45m exercises, the average HR of participants was 111.8 ± 5.3 beats (68% HRmax) and 110.1 ± 8.6 beats (67% HRmax), respectively, and the average HR after high-intensity exercise H10m was 143.0 ± 12.3 beats (86% HRmax). The RPE score differed significantly after the three kinds of exercise. After the M30m and M45m exercises, RPE scores were 13.0 ± 1.8 and 13.1 ± 1.5, respectively. RPE after H10m exercises (16.4 ± 2.3) was higher than in moderate exercises (*p* < 0.01).

## 4. Discussion

This study aimed to analyze the levels of leisure deprivation and quality of life and compare the levels of cortisol, IL-6, and IgA after acute exercise of different intensities and durations to develop an appropriate exercise program for people infected with HIV. The participants recorded high levels of leisure deprivation and moderate quality of life. Further, the immune indices cortisol, sIgA, and IL-6 did not show significant time–group interaction effects. There were varying tendencies for changes in cortisol levels according to the exercise group.

In this study, the highest degree of resource deprivation was observed among the subfactors of relative leisure deprivation, and quality of life exhibited low levels in both life satisfaction and life expectancy. In general, people experience relative deprivation when there is a discrepancy between what they desire and what they actually possess [34]. Various leisure-related public infrastructures and services are collectively built in a certain area and tend to be unevenly distributed depending on the area in Korea [35]. In other words, access to necessary leisure resources and opportunities is relatively limited in small- and medium-sized cities, rural areas, or regional conditions, excluding large cities [36]. People infected with HIV tend to experience relative deprivation of leisure due to a lack of leisure resources in residential areas. Therefore, investment should be directed toward the expansion of facilities and infrastructure so that socially disadvantaged people can freely participate in leisure activities without being alienated from them.

There are several factors that affect the quality of life, and the level of health, such as the fear of disease, is closely related to the quality of life. Indeed, previous studies reported that a significantly lower quality of life is associated with a more negative patient-perceived health status [37,38]. Accordingly, the degree of awareness of one’s mental and physical health affects the quality of life, and most patients with HIV/AIDS suffer from various problems that potentially affect their quality of life from mental, social, and physical health perspectives, such as stigma, poverty, depression, and substance abuse [39]. To prevent negative factors, such as decreased quality of life among people living with HIV, HIV infection prevention should be prioritized. Nonetheless, it is necessary to create an environment wherein individuals can actively participate in mental and physical treatment to improve their quality of life after HIV infection [40]. In addition, the low income and education levels of the participants in this study might have negatively affected their quality of life [41]. Income level was significantly correlated with all areas of quality of life; lower income and education levels resulted in lower health-related quality of life, and quality of life increased with income level [42]. Therefore, it is necessary to apply appropriate economic and public health policies, including raising basic benefits and providing more education and employment opportunities, to improve the quality of life of people living with HIV.

Changes in cortisol, IL-6, and Ig-A levels were analyzed to evaluate the effects of exercise on immune and endocrine functions. These variables can be analyzed through blood sampling; however, variable analysis using saliva samples has recently become an emerging trend because of concerns raised by collectors regarding HIV infection and by participants regarding the invasive nature of blood collection. Cortisol, the principal glucocorticoid in humans, plays a major role in immune function and is used as a biomarker to verify stress levels [43]. In previous studies, increased cortisol levels were observed after exercise compared to those before exercise due to the stress of the exercise stimulus [44,45,46]. However, cortisol levels decreased in all groups immediately after exercise and further decreased after 3 h of exercise compared with pre-exercise levels in the current study. The difference in these results can be attributed to the participants’ stress levels during exercise. Most participants rarely exercised as part of their daily lives, and this was their first time exercising on a treadmill. Low levels of psychological stress in daily life support this perspective. In other words, higher cortisol levels before exercise appear to be due to the fear of performing exercise on the treadmill in participants with low stress in daily life. The results of previous studies suggest that an increase in psychological stress is closely related to an increase in cortisol levels [47]. In addition, the level of secretion is affected by the type, intensity, and duration of exercise [48,49]. In the current study, changes in cortisol levels differed according to exercise intensity and duration.

IL-6 is a pleiotropic cytokine with a wide range of functions. IL-6 can regulate the growth and differentiation of various cells, immune responses, acute responses, and hematopoietic functions, and it plays an important role in the body’s anti-infection immune response [50,51]. Low IL-6 levels in people infected with HIV are important because IL-6 levels are associated with the development of disease and mortality [52,53,54]. In the current study, the time–intensity interaction and main effects of time and intensity were not statistically different in terms of IL-6 changes, and whether the intensity or time condition exerted a positive effect on inflammatory immune factors could not be determined. These results are consistent with those of previous studies that analyzed the effect of exercise on IL-6. A study by Beigpoor revealed that although IL-6 levels in saliva decreased by 21% at 15 min post-high-intensity interval exercise, compared with pre-exercise levels, there was no significant time or interaction effect [55]. Moreover, according to three meta-analyses across three subgroup comparisons, there was also no significant change in IL-6 biomarkers [10]. Similarly, there was no significant change in inflammatory-factor concentrations after 6 weeks of resistance training [56].

Although there are several reasons underlying the lack of significant changes according to time and conditions, the selection of time points for sample collection could be one of the major reasons for collecting the samples immediately and 3 h post-exercise. Presumably, we might have overlooked the time when the inflammatory markers reached a peak or the optimal time for inflammatory changes in the systemic response to physical stress, and changes at these time points, 1 h, 6 h, or 12 h after exercise, should be considered in future studies. Of course, we suspect that the initial kinetics of saliva may be different from the systemic response to these cytokines, and temporal changes in immune response cytokines should be fully considered in future studies. Furthermore, the insignificant difference may be due to the different stages of HIV infection and levels of immunity of the participants. IL-6 levels are known to vary among individuals with HIV.

Salivary immunoglobulin is the first line of defense against the colonization and development of pathogens, thus protecting the organism against infection [57,58]. sIgA has great clinical significance for the cardiovascular, immune, and neuroendocrine systems; sIgA measurement is the mainstay of exercise immunology, and it is used to assess the impairment of humoral immunity by exercise [59]. The results of the analysis of the effect of acute exercise on changes in sIgA levels are controversial [60,61,62]. Differences in IgA changes according to exercise are related to participant characteristics [55]. To the best of our knowledge, no study has analyzed the effect of acute exercise on changes in sIgA levels in patients with HIV. sIgA levels did not change according to exercise intensity or duration in the current study. These results can be attributed to the specificity of the participants. Changes in sIgA levels after acute exercise differ according to the level of fitness [62], and there seems to be no change in exercises of different intensities and durations because of the unlikelihood of sIgA changing with HIV-induced decreases in immune function and physical fitness. However, it is necessary to conduct regular exercise training to improve the immune system, considering the increase in sIgA levels after exercise training in patients with HIV [63].

Some study limitations, such as sample size and saliva collection time after exercise, need to be highlighted. Twelve individuals were initially recruited; however, three participants dropped out during the study, and nine were eventually enrolled. It is not easy to recruit HIV-infected individuals as research participants in Korea because information about them is undisclosed. Therefore, there has been no research on the effect of exercise on HIV-infected individuals in Korea. The study sample size (*n* = 9) is not statistically sufficient to ensure the credibility of the research. Power analysis indicated that at least 18 participants are required for reliable statistical analysis results based on the study design. In addition, saliva was collected from participants three times: before exercise, immediately after exercise, and 3 h after exercise. Perhaps the time of saliva collection after exercise was misjudged or had not reached the response time of inflammatory factors in HIV/AIDS individuals. Hence, future studies involving a sufficient number of participants with more saliva collection times, at least several days for subsequent studies, are required to improve the credibility of the study findings.

However, as a preliminary study to address the problems associated with the methods and procedures in formal research, such as the recruitment of participants, addition or adjustment of factor variables, saliva sample collection time, acquisition of clues or insights such as viewpoints and methods conducive to formal experiments, inspection statistics, and analysis steps, among others, this pilot study provides a basis for a subsequent well-organized study.

## 5. Conclusions

The education and economic levels of the participants were low, without the opportunity for full-time employment. Considering the high level of leisure deprivation and normal quality of life, people infected with HIV could not lead a happy and fulfilling life. However, exercise was shown to be a safe and beneficial intervention in treating people living with HIV/AIDS [26], which could have resulted from the protective effects of exercise on inflammation [64,65,66]. Both moderate and high-intensity exercises result in significant improvements in physical function, endurance, and strength, and may delay the onset of disability and frailty [67].

In the current pilot study, although there were no changes in IL-6 and sIgA levels with different exercise intensities and durations, in the actual experiments, participants showed signs of discomfort after high-intensity exercise. Following the experiment completion, participants expressed that although the time of high-intensity exercise was short compared to that of moderate-intensity exercise, high-intensity exercise caused more physical and mental fatigue and greater pressure than moderate-intensity exercise. In addition, moderate-intensity exercise reduces the discomfort and fatigue associated with high-intensity exercise and improves exercise participation [24]. In summary, considering the small number of participants of the current pilot study, caution should be exercised in interpreting the results. Nevertheless, the preliminary data and participants’ responses to exercises indicate that moderate-intensity exercise should be recommended to improve the health and quality of life of people infected with HIV.

## Figures and Tables

**Table 1:** Individual characteristics of the whole study group (N = 9).

	Information	Mean ± SD/N (%)
Baseline characteristics	Age (years)	54.6 ± 10.8
	Height (cm)	172.1 ± 7.5
	Weight (kg)	78.6 ± 15.2
	HR_max_ (bpm)	165.4 ± 10.8
	BMI (kg·m^−2^)	26.3 ± 3.8
Marital status	Single	7 (77.8%)
	Married	-
	Widowed	-
	Divorced	2 (22.2%)
Children	Yes	1 (11.1%)
	No	8 (88.9%)
Occupation	Entrepreneur	-
	Public servants	3 (33.3%)
	Self-employed	1 (11.1%)
	Students	-
	Unemployed	5 (55.6%)
Census register	Rural	6 (66.7%)
	Urban	3 (33.3%)
Education	Middle school graduate or lower	2 (22.3%)
	High school graduate	4 (44.4%)
	College or higher	3 (33.3%)
Monthly household income	<$800	7 (77.8%))
	≥$800	2 (22.2%))
Duration of infection (years)	0–5	1 (11.1%)
	6–10	3 (33.3%)
	≥11	5 (55.6%)
Infection stage	Asymptomatic	6 (66.7%)
	Mild symptoms	1 (11.1%)
	Advanced Symptoms	2 (22.2%)
	Severe symptoms	-

HR_max_: maximum heart rate; BMI: body mass index; SD: standard deviation.

**Table 2:** Descriptive statistical analysis of relative leisure deprivation.

Variable	Category	Minimum	Maximum	Mean ± SD
Relative leisure deprivation	egoistical deprivation	3.00	4.33	3.56 ± 0.50
	resource deprivation	3.00	5.00	4.00 ± 0.67
	cognitive deprivation	3.00	5.00	3.70 ± 0.69
	emotional deprivation	2.00	5.00	3.22 ± 0.89

SD: standard deviation.

**Table 3:** Descriptive statistical analysis of quality of life.

Variable	Category	Minimum	Maximum	Mean ± SD
Quality of life	physical satisfaction	1.75	4.50	3.02 ± 0.94
	psychological well-being	2.20	4.20	3.22 ± 0.72
	independent satisfaction	2.25	4.25	3.11 ± 0.60
	social relational satisfaction	1.75	4.00	3.02 ± 0.72
	environmental satisfaction	1.88	3.88	3.02 ± 0.57
	spiritual satisfaction	2.50	4.75	3.66 ± 0.82

SD: standard deviation.

**Table 4:** Descriptive statistical analysis of stress.

Variable	Category	Minimum	Maximum	Mean ± SD
Stress	Burnout	1.00	3.90	2.63 ± 0.96
	Depression	1.00	4.00	2.72 ± 1.07
	Anger	1.00	3.75	2.44 ± 0.84

SD: standard deviation.

**Table 5:** Immune indexes of participants before and after different intensity exercise.

Variable	Time	Pre	Post	Post 3 h
Cortisol (μg/dL)	M30m	0.88 ± 0.69	0.72 ± 0.64	0.64 ± 0.51
	M45m	0.84 ± 0.57	0.61 ± 0.42 *	0.52 ± 0.45
	H10m	0.81 ± 0.59	0.79 ± 0.65	0.63 ± 0.52 *
IL-6 (pg/mL)	M30m	4.84 ± 2.44	5.01 ± 2.31	5.24 ± 1.21
	M45m	5.44 ± 1.98	5.71 ± 1.98	4.96 ± 1.84
	H10m	4.35 ± 1.50	5.29 ± 1.82	6.51 ± 2.16
sIgA (μg/mL)	M30m	353.36 ± 72.76	352.41 ± 58.78	333.60 ± 76.85
	M45m	311.72 ± 99.12	307.20 ± 61.20	285.18 ± 80.69
	H10m	327.71 ± 76.19	332.12 ± 47.31	308.33 ± 88.24

Values are presented as mean ± standard deviation. IL6: Interleukin- 6; sIgA: saliva Immunoglobulin A; M30m: moderate-intensity exercise for 30 min; M45m: moderate-intensity exercise for 40 min; H10m: high-intensity exercise for 10 min. *: *p* < 0.05, significantly different from pre-exercise.

## Data Availability

The data presented in this study are available on request from the corresponding author.
